# Identification and developmental expression profiling of putative alkaloid biosynthetic genes in *Corydalis yanhusuo* bulbs

**DOI:** 10.1038/srep19460

**Published:** 2016-01-18

**Authors:** Dengqun Liao, Pengfei Wang, Chan Jia, Peng Sun, Jianjun Qi, Lili Zhou, Xian’en Li

**Affiliations:** 1 Institute of Medicinal Plant Development, Chinese Academy of Medical Sciences & Peking Union Medical College, Beijing 100193, PR China; 2 China Pharmaceutical University, Nanjing 210009, China

## Abstract

Alkaloids in bulbs of *Corydalis* (*C.*) *yanhusuo* are the major pharmacologically active compounds in treatment of blood vessel diseases, tumors and various pains. However, due to the absence of gene sequences in *C. yanhusuo*, the genes involved in alkaloid biosynthesis and their expression during bulb development remain unknown. We therefore established the first transcriptome database of *C. yanhusuo* via Illumina mRNA-Sequencing of a RNA composite sample collected at Bulb initiation (Day 0), early enlargement (Day 10) and maturation (Day 30). 25,013,630 clean 90 bp paired-end reads were *de novo* assembled into 47,081 unigenes with an average length of 489 bp, among which 30,868 unigenes (65.56%) were annotated in four protein databases. Of 526 putative unigenes involved in biosynthesis o f various alkaloids, 187 were identified as the candidate genes involved in the biosynthesis of benzylisoquinoline alkaloids (BIAs), the only alkaloid type reported in *C. yanhusuo* untill now. BIAs biosynthetic genes were highly upregulated in the overall pathway during bulb development. Identification of alkaloid biosynthetic genes in *C. yanhusuo* provide insights on pathways and molecular regulation of alkaloid biosynthesis, to initiate metabolic engineering in order to improve the yield of interesting alkaloids and to identify potentially new alkaloids predicted from the transcriptomic information.

*Corydalis yanhusuo* W.T. Wang, a perennial herb, is in Family *Papaveraceae*. Its dried bulbs are widely used in traditional Chinese medicines to promote blood circulation, reinforce vital energy, inhibit cancer cell proliferation^1^ and to alleviate pains as an analgesic agent (Pharmacopoeia of the People’s Republic of China (2005 edition)). Among hundreds of isolated metabolites including fatty acids, organic acids, amino acids, sugars, steroids, anthraquinones and volatile compounds^2^,^3^,^4^,^5^,^6^, alkaloids of *C. yanhusuo* are known to be its major pharmacologically active compounds in treatment of blood vessel diseases, tumors and various pains^7^,^8^,^9^. The main specifically active alkaloids, including tetrahydropalmatine (THP), corydaline, protopine, columbamine, berberine, dehydrocorydaline (DHC), tetrahydrocolumbamine (THC) and palmatine were determined by *in-vivo* screening tests on animal model rats or human cancer cells^8^,^10^,^11^,^12^. To date, about 60 alkaloids have been identified from dried *C. yanhusuo* bulbs^6^,^13^,^14^,^15^, and all, except leonticine and coryphenanthrine, belong to benzylisoquinoline alkaloids (BIAs). These known BIAs, based on their chemical structures, were classified into six types including protoberberines, aporphines, protopines, isoquinobenzazepines, benzophenanthridines and bisbenzylisoquinolines^13^. All of these compounds are biosynthesized from tyrosine and have a common basic benzylisoquinoline subunit^16^,^17^ (Fig. 1).

Like most medicinally-used herbals, current intensive studies in *C. yanhusuo* focused on isolation and therapeutic functions of alkaloids of interest. The information on alkaloid biosynthesis in *C. yanhusuo*, including relevant biosynthetic enzymes, genes and their regulation is not available, in part due to the absence of its gene sequences. The successful application of RNA-sequencing technology in non-model plants to provide the transcriptomic information and the identification of nearly 200 alkaloid biosynthetic genes in other species (**PlantCYC9.5**; Table S1) allow us to identify and profile expression of *C. yanhusuo* genes that may participate in alkaloid biosynthesis. In this paper, we used the high-throughput Illumina HiSeq™ 2000 sequencing technology (Illumina,San Diego, CA, USA) to generate the first transcriptome data of *C. yanhusuo* axillary bud-derived bulbs. Candidate genes involved in the biosynthesis of BIAs and other types of alkaloids that haven’t been isolated previously in *C. yanhusuo*, were identified by Tblastn searches of our assembled unigenes using known alkaloid biosynthetic genes as queries. THP and protopine accumulations were increased with development of *C. yanhusuo* bulbs^18^, indicating that their synthesis may be developmentally regulated at the transcript level. Thus, in order to examine molecular regulation of alkaloid biosynthesis during bulb development, transcript abundances of these candidate genes obtained by the Illumina sequencing method were compared in *C. yanhusuo* bulbs at the initiation, enlargement and maturation stages, which showed the significant metabolomic difference^5^.

## Results

### Initiation and development of *C. yanhusuo* W.T. Wang bulb

**Figure 2:**
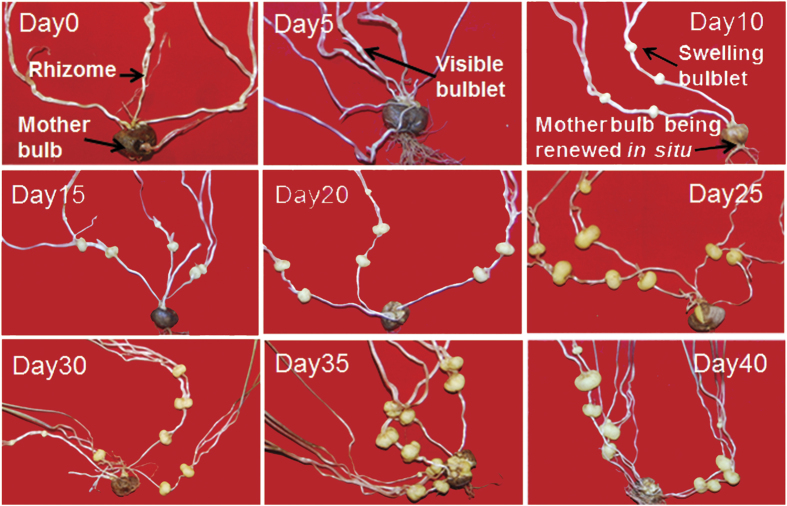
Bulb initiation and development in *C. yanhusuo.* Day 0: Several rhizomes emerged from the mother bulb and no new bulblets were visible; Day 5: new bulblets indicated by a arrow were visible at nodes of rhizomes; Day 10–25: bulbs in enlargement.; 2-3 bulblets were formed on each rhizome. Formation of a new bulb to renew mother bulb *in situ* was simultaneously observed at Day 10; Day 30–40: Bulbs at maturation. Numbers on the images indicated days after the formation of new bulblet initiation, counted from the first sampling.

Bulbs that emerge from axillary buds on horizontally-elongated rhizomes are the major harvested source for farmers. In our greenhouse, the formation of new bulblets started around mid - February and was completed in about 40 days, somewhat faster than in the field. In the field, bulbs develop from early March untill the end May or early June^18^,^19^. As shown in Fig. 2, new white bulblets became visible at 5 days after the first sampling (DAFS), indicating that the differentiation of axillary buds that will develop into new bulbs might already be initiated at the first sampling. New bulbs began visible swelling at about 10 DAFS and completed enlargement at about 25 DAFS. At this stage, new bulbs turned yellow and were about 0.2 cm in diameter. From that stage till 40 DAFS, bulb size did not increase obviously. Based on these morphological observations on bulb development, we divided the 40-day’s developmental process of the *C. yanhusuo* bulb into three stages: bulb initiation (Day 0), bulb enlargement (days 5–25) and bulb maturation (days 30–40). As observed by Liu *et al*.^19^, with the degradation of old scale leaves a new bulb was formed where the mother bulb was located (Fig. 2).

### *De novo* assembly of *C. yanhusuo* bulb transcriptome and its functional annotation

To obtain the first global overview of the *C. yanhusuo* transcriptome, a pooled total RNA sample was prepared from three bulb RNA extracts at bulb initiation (Day 0), early enlargement (Day 10) and at maturation (Day 30) for Illumina paired–end cDNA library construction and sequencing. About 25 million clean paired-end mRNA reads with a mean size of 90 bp and the Q20 percentage of 96.22% were generated and sequentially *de novo* assembled by SOAP into 158,101 contigs, 65,839 scaffolds and 47,081 unigenes ranging from 150 to 7973 bp with the mean length of 489 bp and an N50 length of 681 bp (Table 1). A total of 9,754,893 clean reads, accounting for 39.0% of all raw reads, were uniquely mapped to 47,058 unigenes. Assembly quality analysis revealed that 74.10% of the contigs were shorter than 200b p and only 17,292 scaffolds (26.26%) and 17,274 unigenes (36.69%) were longer than 500 bp (Fig. S1a). However, 79.44% of the scaffolds (52,300) and 98.98% of the unigenes (46,132) had no gap (Fig. S1b).

To reveal the putative functions of the unigenes expressed in *C. yanhusuo* developing bulbs, the BLASTx searches was made against Nr, Swiss-Prot, KEGG and COG protein databases and detected 30,850 protein-coding unigenes (65.53%). Another 2,818 unigenes (5.98%) were identified by ESTScan. The lengths and gap distributions of the predicted protein-coding unigenes were displayed in Fig. S2a,b. Almost all the BLASTx and ESTScan hits had no gap in their aligned or predicted protein-coding regions.

With an e-value cutoff of 10^−5^, the blastx search annotated 65.56% of all unigenes (30,868) to at least one of the above four databases and 7,049 unigenes were annotated to all four (Table 1). 30,660 and 20,664 unigenes were found homologous to the Nr and Swiss-Prot databases, respectively. KEGG analysis enabled 27.85% of the 47,802 unigenes (13,111) to be annotated to the KEGG protein database, of which 9,001 unigenes were mapped by KEGG mapper (ver2.1) to 336 basic reference pathways of 6 modules (updated till Sept. 2014). The six most abundant pathways included metabolic pathways (3203, 24.43%), biosynthesis of secondary metabolites (1730, 13.20%), microbial metabolism in diverse environments (734, 5.60%), plant-pathogen interaction (610, 4.65%), spliceosome (568, 4.33%) and protein processing in endoplasmic reticulum (513, 3.91%) (Table S2). The starch and sucrose metabolism pathway (403, 3.07%) was also found to be dominant in *C. yanhusuo* bulb transcriptome, suggesting starch and sucrose have important roles in bulb development.

The Blastx search against the COG database assigned 9,412 unique unigenes to 25 COG functional categories, amounting to 16,182 COG annotations (Fig. S3). General function prediction only (2,671; 16.51%) was the largest category, followed by transcription (1,430, 8.84%), posttranslational modification, protein turnover and chaperones (1,267, 7.83%), replication, recombination and repair (1,252, 7.74%), translation, ribosomal structure and biogenesis (1,204, 7.44%), carbohydrate transport and metabolism (1,041, 6.43%) and signal transduction mechanisms (1,026; 6.34%).

Of 30,660 Nr-annotated unigenes, 12,455 unique Unigenes had significant GO annotations and were classified into three main GO categories containing 47 broad subcategories (Fig. S4). The biological process category contained 22,360 unigenes and was clustered further into 27 subcategories, which included the top two most abundant subcategories: metabolic process (5,589 unigenes) and cellular process (4,996 unigenes). The cell component category comprised 27,829 unigenes in 11 subcategories including Cell (8,859), Cell part (8,859) and Organelle (6,431). The binding (6,962) and catalytic activity (6503) predominated in the molecular function category containing 15,113 unigenes.

### Overview of differentially expressed genes during *C. yanhusuo* bulb development

To investigate dynamic changes of transcript abundance during bulb development, we performed separate RNA-seq analyses on the bulbs Day 0, Day 10 and Day 30 and evaluated the expression abundances using RPKM-normalized read counts. By means of the Illumina Hiseq 2000 sequencing platform, 6.9–7.5 million single-end (SE) clean reads were generated in the samples (Table 2). Using our assembled *C. yanhusuo* unigenes as the reference transcriptome, 44.8-59.6% of the unique clean reads (3.1–4.1 million) were uniquely mapped, representing 99.8% of the total mapped reads. 45,289, 45,547 and 44,831 reference unigenes were identified in the corresponding individual samples, indicating that our sequencing depth was enough to approach its saturation. The distributions of unigene coverage were similar between the three sampling times, making them comparable (Fig. S5).

**Figure 3:**
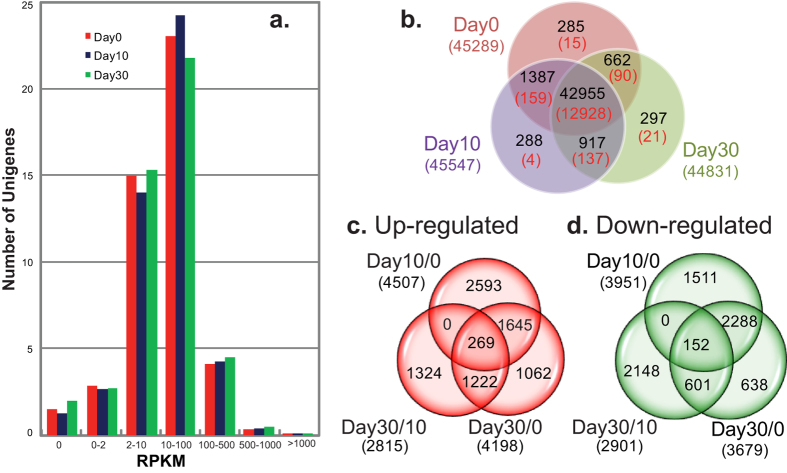
Expression profiling of *C. yanhusuo* Unigenes during the bulb development. (**a**) Number of Unigenes expressed at defined expression levels Numbers on the Y-axis were in units of 1,000. (**b**) Numbers under the stage name are total numbers of DGEs detected by uniquely-mapped reads at the corresponding stage; Numbers in black on Ven diagram meant number of shared and unique genes among the three developmental stages; Numbers in red on the Ven diagram are numbers of shared and unique DEGs among the three developmental stages. a total of 13,354 DEGs were identified in at least one pairwise comparison of developmental stages. (**c,d**) Numbers in () showed are total numbers of DEGs up- or down-regulated between two compared stages. Numbers on Ven diagram are numbers of DEGs shared or unique among different comparisons.

Using only uniquely-mapped SE reads, we detected a total of 46,791 assembled unigenes (99.38%) expressed during *C. yanhusuo* bulb development, of which 46,678 had RPKM ≥2 in at least one developmental stage and 37,943 had RPKM ≥2 at all three studied stages. However, the majority (~80%) were expressed at levels of between 2 and 100 RPKM (Fig. 3a) and 42,955 unigenes were expressed at all three developmental stages (Fig. 3b), 1,387 were co-expressed at Day 0 and Day 10, 917 were co-expressed at Day 10 and Day 30, and 662 were co-expressed in Day 0 and Day 30. About 300 genes were exclusively expressed at one specific developmental stage. With a threshold of FDR ≤ 0.001 and |log2Ratio| ≥1, pairwise developmental stage comparisons revealed 13,354 unigenes with RPKM values ranging from 0 to 24867 and showing expression differences in at least one bulb stage. This represented 28.36% of the bulb transcriptome (Fig. 3b). In detail, 12,928 of 42,955 co-expressed unigenes were differentially regulated during bulb development. In our sequencing depth, 426 Unigenes were specifically expressed at one or two stages. Specifically, 159, 137 and 90 unigenes co-expressed at Day 0 and Day 10, Day 10 and Day 30 and Day 0 and Day 30, respectively; only 15, 4 and 21 unigenes were specifically expressed in Day 0, Day 10 and Day 30, separately. The distribution of total numbers of DEGs up- or down- regulated between any two stages and their relationship among different comparisons were shown in Fig. 3c,d; 4,501 and 3,951 DEGs were up- and down-regulated separately at Day 10 compared to Day 0. A similar number of DEGs were differentially expressed between Day 30 and Day 0. The comparison of Day 30 and Day 10 identified 2,815 enhanced DEGs and 2,901 decreased DEGs, less than the earlier two comparisons, indicating a lower number of genes showing differential expression from bulb enlargement till maturation. Expression abundances of only 269 and 152 DEGs were significantly increased or decreased by at least 2 folds throughout bulb development, indicating that most transcripts were transiently significantly regulated at different stages of bulb developmental stages. For example, of 4,507 enhanced unigenes during the Day 0 to Day 10 comparison, 2,593 unigenes were increased only at Day 10, and 1,645 unigenes overlapped with the Day 30 to Day 0 comparison. This indicated that they had remained at high expression levels at Day 30 following increases detected at Day 10, but there was no quantitative difference between the two sampling times (Fig. 3c).

### Functional analysis of differentially expressed genes

Figure S6 showed the distribution of developmentally modulated transcripts in 24 COG functional categories. Besides General function prediction only, Carbohydrate transport and metabolism and Transcription were the next two prominent categories in both the Day 10/0 and Day 30/0 comparisons. In the Day 30/10 comparison, Transcription and Posttranslational modification, protein turnover, chaperones were dominant.

KEGG pathway analysis revealed a total of 3,209 DEGs, accounting for 69.03% of all KEGG-annotated DEGs. These were assigned to 276 basic reference pathways of five modules, among which metabolic pathways and biosynthesis of secondary metabolites dominated in all of three comparisons (Table S3). Sixty-eight, 26 and 59 pathways were enriched in Day 10/0, Day 30/10 and Day 30/0, respectively (Table S4), among which, there were 19 common enriched KEGG pathways, including Biosynthesis of secondary metabolites, Starch and sucrose metabolism, Glycolysis/Gluconeogenesis, Ascorbate and aldarate metabolism in carbohydrate metabolism and Tyrosine metabolism and Phenylalanine metabolism in the amino acid metabolism group. Phytohormones such as CK, auxin and GA and the underlying genes have important roles in the initiation and development of plant organs^20^,^21^,^22^. Unigenes involved in Plant hormone signal transduction were also significantly enriched in the comparisons of Day 10/0 and Day 30/0, suggesting that phytohormones play important roles in regulating the initiation and enlargement of bulbs and should be a future topic for research. GO analysis revealed that biological processes of the DEGs including carbohydrate metabolic process (GO:0005975) and response to stimulus (GO:0050896) were mainly enriched in Day 10/0 and Day 30/0 (Table S5), indicating that the bulb enlargement and maturation was associated with storage metabolism and stimulus signaling.

### Putative alkaloid biosynthetic unigenes identified by KEGG-mapping analysis

Transcriptome-wide KEGG pathway analysis showed that 1,730 unigenes were involved in biosynthesis of secondary metabolites in *C. yanshusuo* bulbs (Table S2). Among them, 72 were annotated to participate in alkaloid biosynthesis (Table S6). Of the five assigned alkaloid biosynthetic pathways, isoquinoline alkaloid biosynthesis (49 unigenes) followed by Tropane, piperidine and pyridine alkaloid biosynthesis (30 unigenes) and Indole alkaloid biosynthesis (18 unigenes) was well represented in the transcriptome. This was consistent with the main types of abundant alkaloids found in *C. yanhusuo* bulb extracts, such as glaucine, tetrahydropalmatine, dehydrocorydaline and palmatine^14^,^23^. Isoquinoline alkaloid biosynthesis was enriched in all bulb stages and 34 unigenes in this pathway were regulated by bulb developmental stage (Table S4). However, it should be noted that some KEGG-identified unigenes may participate indirectly in alkaloid biosynthesis, functioning well upstream pathway in the alkaloid biosynthesis pathways, or as a linkage between primary and secondary metabolisms. For example, aspartate aminotransferase was associated with the biosynthesis of tyrosine-derived isoquinoline alkaloids, tropane, piperidine and pyridine alkaloids, because the product, aspartate, can participate in the biosynthesis of tyrosine as an indirect precursor^24^. To complement KEGG mapping results, we further examined the presence and expression of *C. yanhusuo* unigenes homologous to known alkaloid biosynthetic genes (Table S1).

### Candidate genes with homology to known BIAs biosynthetic genes

**Figure 4:**
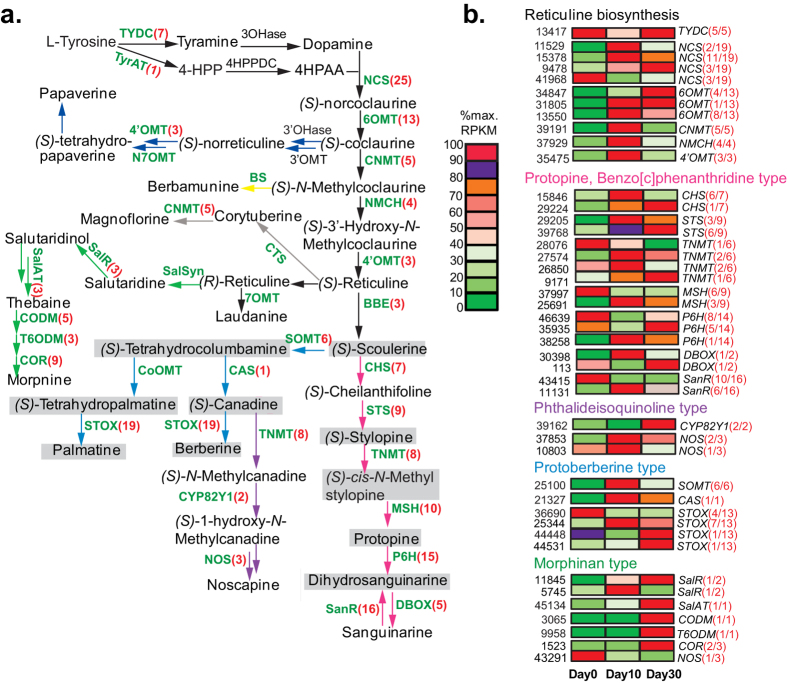
Expression patterns of BIAs biosynthetic unigenes during *C. yanhusuo* bulb development. (**a)** Biosynthetic pathways leading to major benzylisoquinoline alkaloids (BIAs) are based on Beaudoin and Facchini^26^. Pathways: black arrows 1-benzylisoquinoline; dark blue arrows, papaverine; green arrows, morphinan; purple arrows, phthalideisoquinoline; light blue arrows, protoberberine; yellow arrows, bisbenzylisoquinoline; grey arows, aporphine; pink arrows, benzo[c]phenanthridine; Enzymes for which corresponding genes have been isolated from other plants were shown in green. Enzymes for which corresponding genes have not been isolated from any plant are shown in black. The number of identified *C. yanhusuo* unigenes is shown in () following the abbreviated nameof the corresponding enzyme whose full name can be found in Table S1. Intermediates or alkaloid compounds that have been identified in *C. yanhusuo* are shaded in gray. Although coptisine, tetrahydrojatrorrhizine, jatrorrhizine, allocryptopine, N-methyltetrahydrocolumbamine, N-methyltetrahydrocoptisine and columbamine are also present in *C. yanhusuo* bulbs, their final biosynthetic steps mainly involved STOX^28^ and are not displayed here since STOX catalyzes the formation of many such metabolites. Two unigenes identified as Pavine N-methyltransferase are not listed here. Abbreviations unlisted in Table S1: 4HPP, 4- Hydroxyphenylpyruvate; 4HPAA, 4- Hydroxy- phenylacetaldehyde; *3OHase* tyrosine/tyramine 3-hydroxylase; *4HPPDC* 4-hydroxyphenylpuruvate decarboxylase; *3*′*OHase* uncharacterized 3′-hydroxylase; *3*′*OMT* uncharacterized 3′-*O*-methyltransferase. (**b**) Expression patterns of representative DEGs. Only one of the differentially expressed BIA unigenes with similar expression patterns is shown here to represent different expression patterns of the gene family. The grids with 10 different colors from dark green to red show the relative expression levels to maximum RPKM values: 0–10, 10–20, 20–30, 30–40, 40–50, 50–60, 60–70, 70–80, 80–90 and 90–100%, respectively. The detailed value for each gene is given in Table S7. Unigene IDs are shown on the left. On the right of the grids, the numbers of differentially expressed BIA unigenes and DEGs with similar expression patterns are shown in (), following the abbreviated names of the corresponding enzymes. Expression of the enzymes functioning in multiple branch pathways is displayed just once.

Most of the reported alkaloids in *C. yanhusuo* are BIAs^13^. The biosynthesis of BIAs including the routes and enzymes involved in the synthesis of each one is relatively clear, although not complete^25^,^26^,^27^ (Fig. 4a). Among the known intermediates /end-products in BIAs biosynthesis pathways, only a few have been identified in *C. yanhusuo* bulbs, as shaded in grey in Fig. 4a. However, homologs of almost all the known enzymes were identified in the *C. yanhusuo* bulb transcriptome (Table S7, Fig. 4a), indicating that BIA biosynthesis among different BIA-producing plant species shared most of common steps, especially those in the upstream pathways. For example, (S)-reticuline is the key intermediary branch-point in biosynthesis of different types of BIAs and exists in a varied abundance in many BIAs-producing plants^28^. Homologs that we failed to identify in *C. yanhusuo* are mainly downstream in the BIAs biosynthesis pathway, and include reticuline 7-O-methyltransferase (7OMT), norreticuline 7-O-methyltransferase (N7OMT), berbamunine synthase (BS), corytuberine synthase (CTS), salutaridine synthase (SalSyn) and columbamine *O*-methyltransferase (CoOMT). The majority of functionally characterized BIAs biosynthetic genes (Table S1) were cloned from opium poppy (*papaver somniferum*), which, like *C. yanhusuo,* belongs to family Papaveraceae and is thought to be the only source of morphine, codeine, sanguinarine and papaverine^29^. This may indicate that synthesis of the substrates /alkaloids by the downstream enzymes (BS, CTS, N7OMT and 7OMT) are themselves species-specific and deficient or undetectable in *C. yanhusuo*. However, this needs to be further biochemically investigated in *C. yanhusuo*.

DEG analysis revealed that BIAs biosynthetic unigenes were highly regulated during bulb development (Table S7, Fig. 4b). Among 187 putative BIA biosynthetic unigenes, 147 encoding 24 enzymes except for tyrosine aminotransferase and berberine bridge enzyme were differentially expressed across the three studied developmental stages. One hundred and fourteen DEGs were significantly up-regulated durign bulb development, reaching their maximum expression levels at early bulb enlargement (Day 10) or at maturation (Day 30) (Fig. 4b). These up-regulated unigenes were distributed in all 24 gene families, indicating that biosynthesis of various BIAs is synchronously regulated in both the early route (reticuline biosynthesis) and the late steps after branch points. In addition, 33 DEGs from gene families encoding NCS, STOX, TNMT, MSH, P6H, SanR, and COR were highly expressed at bulb initiation but less at the later stages. However, some members in these gene families underwent the increased expression with bulb development. Five DEGs of TYDC which yields tyramine or L-DOPA for tyrosine-derived BIA biosynthesis were down-regulated at bulb enlargement and then increased at maturation, showing a different expression pattern from other BIA biosynthetic DEGs. This difference may indicate that expression of TYDC is regulated by multiple factors since its products tyramine or L-DOPA serve not only as the distant precursors to tyrosine-derived BIA biosynthesis but also as immediate precursors to various amines and amides.

### Candidate genes for non-isoquinoline-type alkaloids biosynthesis in *C. yanhusuo* bulbs

**Figure 5:**
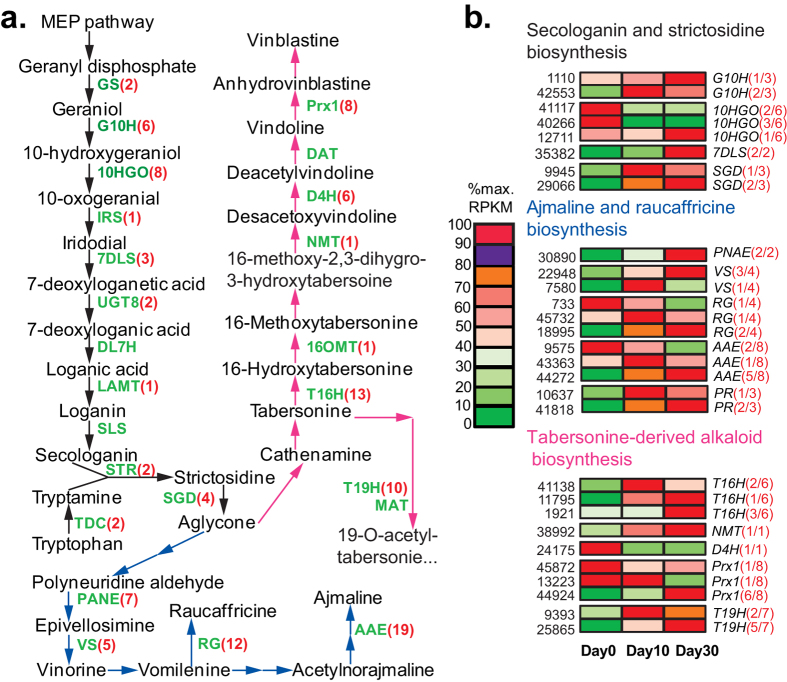
Expression patterns of Monoterpenoid Indole Alkaloids (MIAs) biosynthetic unigenes during bulb development. (**a**) Biosynthetic pathways of MIAs are based on PlantCYC (9.5), Gidding *et al*.^52^, De Luca *et al*.^53^ and Verma *et al*.^54^. Enzymes for which corresponding genes have been isolated from other plants are shown in green next to the arrows. Two or more known steps between two intermediates, whose cDNA sequences were not isolated, are omitted here but indicated by two continuous arrows. Black arrows, secologanin and strictosidine biosynthesis; blue arrows, ajmaline and raucaffricine biosynthesis after the branch-point intermediate aglycone; pink arrows, tabersonine-derived alkaloid biosynthesis after the branch-point intermediate aglycone, represented by vindoline and vinblastine biosynthesis. Full names of enzymes represented by their abbreviated names can be found in Table S1. (**b**) Expression patterns of representative DEGs. Illustrations are provided in Fig. 4b.

**Figure 6:**
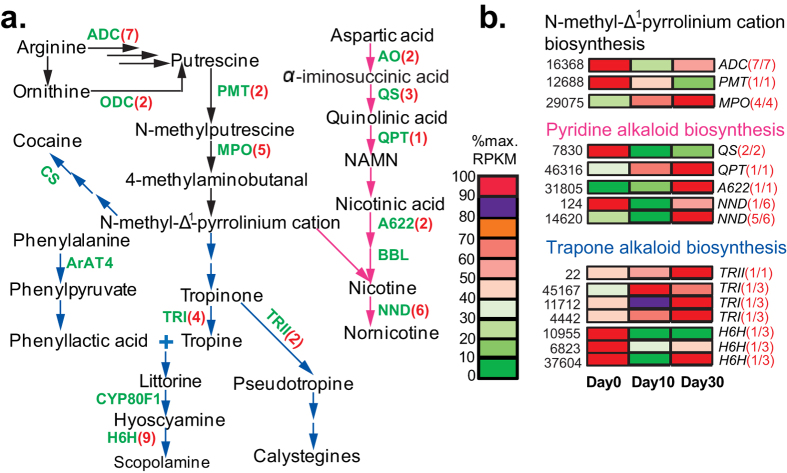
Expression patterns of Tropane and Pyridine alkaloids biosynthetic unigenes over *C. yanhusuo* bulb development. (**a**) Biosynthetic pathways are based on Dewey and Xie^55^, Kushwaha *et al*.^56^ and Bedewitz *et al*.^57^. Black arrows, N-methyl-Δ^1^-pyrrolinium cation biosynthesis; blue arrows, various tropane alkaloids biosynthesis; pink arrows, pyridine alkaloid biosynthesis, represented by nicotine biosynthesis. Enzymes for which corresponding genes have been isolated from other plants are shown in green next to the arrows. Two or more known steps between two intermediates, whose cDNA sequences haven’t been isolated, are omitted here but indicated by continuous arrows. Full names of enzymes by abbreviations are provided in Table S1. NAMN, nicotinic acid mononucleotide. (**b**) Expression patterns of representative DEGs. Illustrations are provided in Fig. 4b.

**Figure 7:**
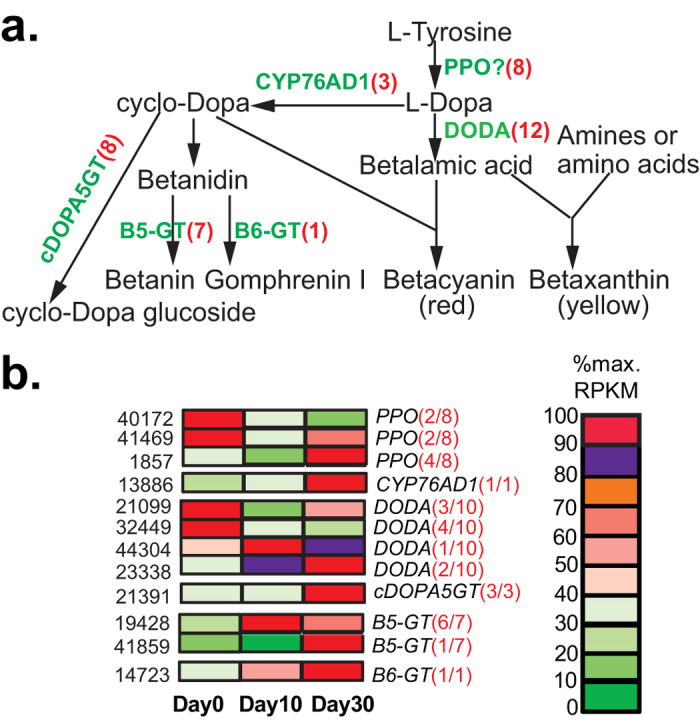
Expression patterns of betalain alkaloids biosynthetic unigenes duringbulb development. (**a**) The betalain biosynthetic pathway is modified from Suzuki *et al*.^58^. Full names of abbreviated enzymes can be found in Table S1. (**b**) Expression patterns of representative DEGs. Illustrations are provided in Fig. 4b.

Hither to, no phytochemical work has been conducted to determine whether *C. yanhusuo* contains other types of alkaloids such as indoles and tropane alkaloids. Although some of these types are mainly or specifically found in certain plant families, several indole alkaloids were isolated from the isoquinoline-producing plants *Corydalis saxicola*^30^ and papaveraceous species^31^, indicating that non-isoquinoline alkaloids may also be synthesized in *C. yanhusuo*. Furthermore, KEGG analysis identified the involvement of some *C. yanhusuo* unigenes in biosynthesis of non-isoquinoline-type alkaloids (Table S6). Therefore, we also examined the presence and expression of putative *C. yanhusuo* homologs involved in the biosynthesis of non-isoquinoline-type alkaloids including indole alkaloids (Fig. 5), Trapone and Pyridine alkaloids (Fig. 6) and Betalains (Fig. 7) (full information is provided in Table S8. Known genes for the indole alkaloid biosynthesis, which were mainly isolated from *Catharanthus roseus* (Table S1). participate in the biosynthesis of monoterpenoid indole alkaloids (MIAs). Through the two branch pathways (terpenoid-forming and indole pathways), the upstream MIAs biosynthesis pathway synthesizes strictosidine, the common final precursor for various downstream MIAs (Fig. 5a). By sequence similarity searches, we identified homologs of all the known MIAs genes in the *C. yanhusuo* transcrtiptome, except for 7-deoxyloganic acid-7-hydroxylase (DL7H) and secologanin synthase (SLS) involved in the strictosidine biosynthesis and downstream MIAs biosynthetic genes including two O-acetyltransferase genes (Deacetylvindoline-4-O-acetyltransferase (DAT) and Minovincinine-19-hydroxy-O-acetyltransferase(MAT)), Tetrahydroalstonine synthase and N-methyl-3-aminomethylindole N-methyltransferase (Table S8, Fig. 5a). DL7H and SLS are important enzymes in the terpenoid-forming pathway that synthesizes secologanin. Both belong to the CYP72A subfamily and share about 50% sequence identity^32^. By tBlastn searches, we identified three *C. yanhusuo* unigenes sharing 40% identity with DL7H and SLS, but belonging to CYP734A subfamily (data not shown). Identification of intermediates /metabolites such as secologanin in the future will further clarify whether *C. yanhusuo* bulbs synthesize secologanin-derived MIAs and confirm the involvement of related biosynthetic genes identified in this study. We were able to identify the *C. yanhusuo* homologs for 12 of 17 known trapone and pyridine alkaloid biosynthetic genes and all 7 known betalaine biosynthetic genes in our transcriptome (Figs 6 and 7). Expression analysis revealed that developmentally regulated MIAs biosynthetic unigenes were mainly found in downstream synthesis routes of specific MIAs (Fig. 5b). In upstream secologanin and strictosidine biosynthesis, only four gene families, including geraniol 10-hydroxylase, 10-hydroxygeraniol oxidoreductase, 7-deoxyloganetic acid synthase and strictosidine glucosidase had differentially expressed genes across the three developmental stages. Similar to BIAs biosynthetic unigenes, expressions of gene families involved in biosynthesis of trapone and pyridine alkaloids and betalaine alkaloids were collectively regulated by the bulb development (Figs 6b and 7b).

The representativeness of the known genes participating in the biosynthesis of acridone alkaloid, capsaicinoids, caffeine and steroidal glycoalkaloids in *C. yanhuso* was similar to BIAs biosynthetic genes (Table S8). That is, genes encoding enzymes involved in early or certain biosynthetic steps of these alkaloids or their derivatives were present among *C. yanhuso* unigenes. However, no homologs of the key enzymes especially in the branch-point or final steps of respective alkaloid biosynthesis were found in our current transcriptomic database. This finding is not surprising, because the processes in which the identified genes were involved are not only confined to alkaloid metabolism but are also connected with many other pathways in the plant metabolic network. For example, the intermediates/products of the phenylpropanoid pathway that produces phenylalanine precursor for capsaicinoid biosynthesis^33^ also have roles in synthesis of lignin and phenolics^34^. These unidentified enzymes included anthranilate N-methyltransferase, acridone synthase, four caffeine biosynthetic enzymes, cocaine synthase, capsaicin synthase and so on (Table S8), indicating that *C. yanhusuo* bulbs may not synthesize some kinds of alkaloids isolated in other species. Information on expression of the identified candidate unigenes was shown in Table S8.

### HPLC-PDA analysis

**Figure 8:**
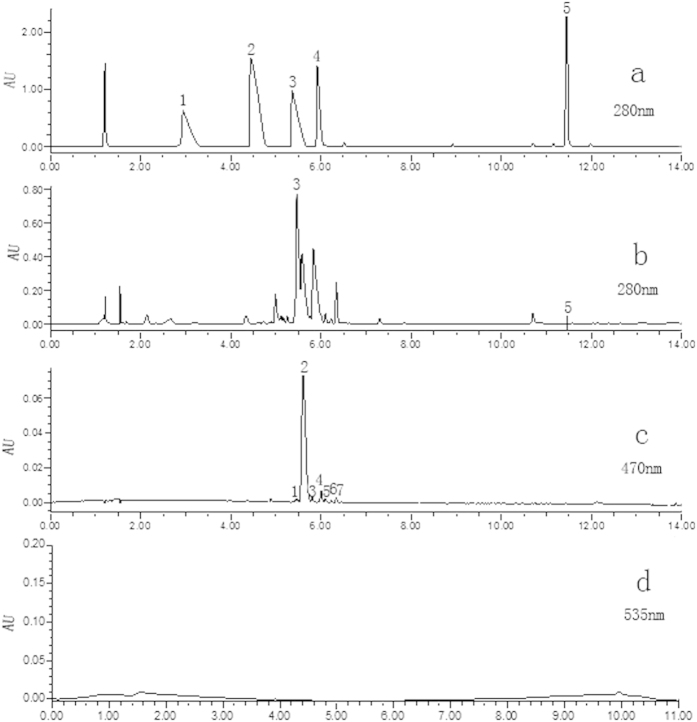
Chromatograms of BIA standards at 280 nm (**a**) and *C. yanhusuo* bulb extract monitored at 280 (**b**), 470 (**c**) and 535 nm (**d**). Numbers in (**a**) and (**b**) refer to magnoflorine (1), corytuberine (2), tetrahydropalmatine (3) sanguinarine (4) and dihydrosanguinarine (5). Numbers in (**c**) refer to unknown components detected at 470 nm.

To justify the feasibility of transcriptomic information in predicting existence or non-existence of new alkaloids in *C. yanhusuo* bulbs, five BIAs, including sanguinarine (SG), dihydrosanguinarine (DHSG), magnoflorine, corytuberine and THP, and betalain-type alkaloids were chosen for biochemical analysis. Except for the previously known THP and DHSG, sanguinarine(SG), magnoflorine and corytuberine were not detected in *C. yanhusuo* bulb (Fig. 8b), Absence of the last two was consistent with the transcriptomic information (Fig. 4a).

Betalains comprise betaxanthins and betacyanins, which respectively have UV- vis wavelength absorbance maxima at around 470 nm^35^ and 540 nm^35^,^36^. HPLC analysis detected several peaks at 470 nm (Fig. 8c); however, further full UV-vis wavelength spectra scanning showed that the absorbance maxima were between 200 and 360 nm, rather than 470 nm (Fig. S7), indicating that no betaxanthins were present in *C. yanhusuo* bulb, the same as betacyanins (Fig. 8d). This result conflicted with our earlier transcriptomic analysis that revealed that *C. yanhusuo* contained and expressed homologs of all 7 known betalaine alkaloids biosynthetic genes (Fig. 7). This suggested that one or more step(s) in the betalain biosynthetic pathway was inactivated in *C. yanhusuo*. Recently, betalains synthesis was induced in non-betalain plants or anthocyanin-producing species via L-DOPA feeding^37^,^38^ and/or the introduction of a betalain-specific tyrosine-hydroxylating gene and a 4,5-dioxygenase (DODA) gene from betalain-producing plants^38^,^39^. These studies demonstrated that hydroxylation of tyrosine to DOPA, and the conversion of L-DOPA to betalamic acid were crucial steps for successful betalain synthesis. Moreover, they indicated that non-betalain, anthocyanin-producing plant species including Arabidopsis and potato lack the tyrosine-hydroxylating enzyme and DOPA enzymes that are essential for betalain biosynthesis. Phylogenetic analyses of CYP76AD1 and DODA among betalainic taxa and anthocyanic taxa of the order Caryophyllale revealed that their isoforms had diverged into betalain-specific clades and anthocyan-specific clades during the evolution^40^. Anthocyanic taxa lack or have decreased DODA-α and CYP76AD1-α isoforms that catalyze L-DOPA into betalamic acid and cyclo-DOPA, respectively. Thus, no or only traces of betalains are synthesized in anthocyan-producing plants. Although sequence similarity comparison assigned 15 *C. yanhusuo* unigenes as homologs of the known betalain-specific CYP76AD1 and DODA genes (Fig. 7 and Table S8), it is possibly the same reason for no betalains detected in *C. yanhusuo* bulb.

## Discussion

Palmatine and its derivated THP are the main bioactive alkaloids in *C. yanhusuo* bulb. Their final biosynthetic steps involve columbamine *O*-methyltransferase (CoOMT) and (*S*)-tetrahydroprotoberberine oxidase (STOX) (Fig. 4a). Although CoOMT has been shown to participate in palmatine biosynthesis in *Berberis wilsoniae* and *Berberis aggregate* in family Berberidaceae^41^ and in *Coptis japonica* of family Ranunculaceae^42^, however, the cDNA sequence encoding CoOMT was cloned only from *Coptis japonica* cultured cells^42^. Tblastn of CjCoOMT combining Nr annotation find no CoOMT homologs among our *C. yanhusuo* unigenes (Table S7, Fig. 4a). Several reasons could account for the failure to detect homologs of a known gene in our transcriptome database: i. low expression making it hard to detect. ii. the gene is temporally and spatially- specific and not expressed in our sampled tissue and stage; or iii. protein sequence variation between different species. We excluded the first two reasons, because THP and palmatine were the second and fourth most abundant BIAs in the bulbs^23^ and therefore genes responsible for their synthesis, especially the key steps, should be highly expressed and detectable. Furthermore, *C. bracteata* BIAs were found not translocated between plant organs and were only *de novo* synthesized in studied organs^43^. In addition, THP accumulated throughout bulb growth^18^. Thus, THP/palmatine biosynthetic genes should be expressed in our samples representing three key stages of bulb development. It is therefore most likely that a protein sequence variant is responsible for non-detection of CoOMT homologs in *C. yanhusuo*. Many reports show that palmatine is widely distributed in *Phellodendron amurense, Berberis* species, *Mahonia* species, *Enantia* species, *Coptis* species, *Stephania* species and *Corydalis* species, all of which belong to different plant families. We further examined the presence of CoOMT homologs in other palmatine-producing medicinal plants via blast searches against their available 454 transcriptomic databases (Table S9). Ours results based on protein sequence similarity showed that CoOMT homologs were present only in plants of core Ranunculales family Ranunculaceae excluding *Xanthorhiza simplicissima*, Berberidaceae and Menispermaceae. They are not detected in early-diverging Ranunculale family Papaveraceae to which *C. yanhusuo* belongs. Among BIA-related OMTs, PsSOMT2 clustered with the same clade as CjCoOMT, 4’OMTs and 6OMTs^44^. PsSOMT2 and its homologs were identified in the early-diverging Ranunculale Papaveraceae. In-vitro enzymatic analysis showed that PsSOMT1 in the same clade as CjSMT, but not PsSOMT2, can convert THC into THP while *O-*methylating its main substrate *(S)-*scoulerine into THC^44^. This suggested that the function of PsSOMT2 homologs and their catalyzed substrates changed accordingly with their protein sequences in the evolution of core Ranunculales from early diverging Ranunculales. There were three *C. yanhusuo* unigenes weakly homologous to PsSOMT1 and PsSOMT2, three unigenes to CjSMT, 13 unigenes annotated as 6OMT and three as 4’OMT (Table S7, Fig. 4a).

Among six morphine-specific biosynthetic enzymes (Fig. 4a), salutaridine synthase (SalSyn) was the only unidentified enzyme in the *C. yanhusuo* transcriptome. SalSyn catalyzes the phenol-coupling reaction. Except for the substrate recognition sites, the overall amino acid composition is poorly conserved with other phenol-coupling enzymes such as CYP80s and the human cytochromes P450 2D6 and 3A4 which catalyze C-C phenol-coupling of reticuline^45^. However, two *C. yanhusuo* unigenes having low homology with *Papaver somniferum* Salsyn protein were either not long enough to cover the conserved substrate recognition site region or lacked conserved amino acids at substrate recognition sites.

## Conclusions

Alkaloids are the major bioactive compounds in *C. yanhusuo* bulbs and so far BIAs are the only identified alkaloid type. Here, for the first time, we presented the transcriptomic information for *C. yanhusuo* bulbs via the next-generation transcriptomic sequencing and bioinformatic analysis. Through blast searches against our transcriptomic database using known alkaloid biosynthetic genes as queries, the majority of BIAs enzymes involved in the biosynthetic pathway had homologs present in *C. yanhusuo* bulbs. Expression analysis showed that the BIAs biosynthetic candidate genes were regulated in the overall pathway during the bulb development. We also identified *C. yanhusuo* unigenes involved in certain steps of biosynthetic pathways of other types of alkaloids including MIAs, tropane alkaloids and betalaine alkaloids. Genes that we failed to identify in *C. yanhusuo* were mainly downstream in the alkaloid biosynthesis pathway and catalyzed the formation of species-specific alkaloids, which clarified at the transcriptomic level why non-isoquinoline-type alkaloids were not detected in *C. yanhusuo* using HPLC-DAD and ESI-MS methods (MS data not shown in the paper). Our results not only provided knowledge on molecular mechanisms regulating the biosynthesis of known alkaloids in *C. yanhusuo* but also information indicating what unknown alkaloids possibly exist in the bulbs and transcriptiomic basis for undetected non-isoquinoline-type alkaloids. Our study will help determine and narrow the range of enzymes to introduce into *C. yanhusuo* in the future genetic engineering.

## Methods

### Plant growth conditions and sampling

Seed bulbs of *C. yanhusuo* W.T. Wang (3 plants/pot) were planted in September in pots (30 × 30 × 20cm) containing soil under glasshouse conditions with natural lighting and temperatures of 15–25 °C at the IMPLAD (Institute of Medicinal Plant Development), Beijing, China. Developing bulbs were harvested for growth observations on growth every 5 days starting from new bulb initiation (Day 0) near the end of following February till mid-April (Day 40).

### RNA extraction, Illumina cDNA library construction and sequencing

Developing bulbs were harvested at Days 0, 10 and 30, frozen immediately in liquid nitrogen and stored at −80 °C untill used. Total RNA was extracted using TRIplant reagent (BioTeke, Beijing, China) following the manufacturer’s instructions, quantified spectrophotometrically at 260 nm and submitted to the Beijing Genomics Institute (BGI, Shenzhen, China) for Illumina mRNA library construction and HiSeq™ 2000 sequencing. In order to obtain the reference transcriptome data for *C. yanhusuo*, equal quantities of high-quality total RNA from the above three bulb stages were pooled for Illumina paired-end cDNA library preparation. For digital expression profiling of genes during bulb development, separate RNA samples from all three stages were used for Illumina single-end cDNA library preparation. Briefly, Illumina mRNA-seq library construction was as follows: mRNA was purified from 20 μg of DNaseI-treated total RNA with a RIN of ≥8 using oligo(dT) magnetic beads and then sheared into short fragments by heat treatment in fragmentation buffer. Cleaved mRNA fragments were reverse transcribed into the first cDNA strands using random hexamer primers followed by the second-strand cDNA synthesis using DNA polymerase I and RNase H. The dscDNAs were purified using a QiaQuick PCR extraction kit (Qiagen), washed with the elution buffer and followed with end repair, Poly (A) tailing addition and sequencing adapter ligation. The ligated products were run on agarose gels and suitable fragments were size-selected for PCR amplification as templates. The PCR libraries were purified, quantified and tested for quality by an Agilent 2100 Bioanalyzer and ABI StepOnePlus Real-Time PCR System and sequenced using Illumina HiSeq™ 2000.

### *De novo* assembly and annotation of the reference transcriptome

The 90 bp paired-end raw reads were generated on the Solexa/Illumina platform to establish a reference transcriptome of *C. yanhusuo*. The clean reads were produced by removing the raw reads containing the adapters, any unknown nucleotides larger than 5% and low-quality reads containing more than 50% bases with Q-values ≤20. Preprocessed reads with a certainty of overlapping lengths were first de novo assembled into gapless contigs (≥60 bp) using the short read assembler–SOAPdenovo (version 1.04) (http://soap.genomics.org.cn/). Using paired-end reads, SOAPdenovo detected the contigs from the same transcripts and joined them into scaffolds (≥100 bp) using Ns to represent unknown bases between adjacent contigs. Unigenes with the lowest numbers of Ns were generated by further using paired-end reads to fill gaps between scaffolds until no further extension could be achieved at either end.

Unigene annotation was performed by BLASTx against protein databases including NCBI non-redundant (Nr), Swiss-Prot, KEGG and Clusters of Orthologous Groups (COG) with an e-value threshold ≤1 × 10^−5^. The best hits together with a priority order of Nr, Swiss-Prot, KEGG and COG, in the case of alignment conflict among the four databases, were used to decide coding sequence regions and direction of unigenes. When a unigene could not be aligned to any one database, ESTScan^46^ was used to predict its coding region and to decide the sequence direction. The Blast2GO^47^ program and WEGO software^48^ were used in unigene GO (Gene Ontology) annotation and functional classification.

### Profiling of differentially expressed genes (DEGs) using mRNA-seq reads

Fifty bp single-end raw reads were generated from mRNA-seq of the three individual developmental stages of *C. yanhusuo* bulbs and preprocessed by removing the reads containing only adaptors, more than 10% of unknown bases (N) and low-quality reads containing more than 50% bases with Q-values ≤5. The clean reads for each stage were then independently mapped onto the *Corydalis* reference transcriptomic assembly using SOAPaligner/soap2 (version2.20)^49^, allowing a maximum of 2 bp mismatches per read. To estimate the expression levels of transcripts, the number of uniquely-mapped reads for each unigene in a given bulb stage was computed and normalized to RPKM (Reads Per kb per Million reads) values using the RPKM formula^50^. If there were multiple unigenes for a gene, the longest one was used to calculate its expression level and coverage.

RPKMs of unigenes with missing values in a specific sample were adjusted to 0.001. RPKM values of unigenes were then compared on a pairwise developmental stage basis, that is, Day 10 versus Day 0, Day 30 versus Day 10 and Day 30 versus Day 0. P-values were calculated using the formula described by Liu *et al*.^51^ to identify genes expressed differentially between paired developmental stages. Significantly DEGs were identified using an FDR (false discovery rate) threshold of ≤0.001 and a minimum two-fold change.

### Functional enrichment analysis of differentially expressed genes

Gene ontology (GO) and KEGG pathway enrichment analyses of DEGs were conducted on the three genesets obtained by pairwise stage comparisons. Hypergeometric distribution test followed by Benjamini-Hochberg correction was applied to assess the significance of each term. The cutoff of Q-values for enriched function was ≤0.05.

### Identification of alkaloid biosynthetic genes in *C. yanhusuo* bulb

*C. yanhusuo* alkaloid biosynthetic genes were decided by a combination of direct screening of transcriptome assembly annotations (Nr and KEGG) and Tblastn searches of the *C. yanhusuo* unigenes using known alkaloid biosynthetic genes (Table S1).

### HPLC-PDA analysis of selected alkaloids

Standards of sanguinarine, dihydrosanguinarine, magnoflorine, corytuberine and tetrahydropalmatine with 98% of HPLC purity were purchased from Herbest Bio-Tech Co. Ltd, China (http://www.herbest.cn/). THP was used for the internal quality control of experimental procedures including sample extraction and HPLC-PDA analysis.

*C. yanhusuo* bulbs at maturation were fast frozen in liquid nitrogen and stored at −20 °C till use. The samples of three biological replicates were homogenized with liquid nitrogen and 200 mg of each replicate was extracted with 1 ml of 80% aqueous methanol (v/v) in an ultrasonic bath for 30 min at 30 °C. The extract was then centrifuged at 10,000 rpm for 10 min at 4 °C. The supernatant was collected, filtered through a 0.45-μm membrane filter and stored at −20 °C for HPLC–PDA analysis. Four microliters of extracts and standards were run at 25 °C on a Waters Acquity ultra performance liquid chromatography (UPLC, Ireland) system coupled with a Waters BEH C18 column (1.7 μm, 2.1 × 100 mm) and a photodiode array detector. The mobile phase consisted of acetonitrile (A) and 0.2% formic acid in water (B) and was delivered at 0.2 ml/min. A linear gradient was used as follows: 0–4 min, 20% A; 4–11 min, A from 20 to 100%; 11–14 min, A from 100% to 20%. DAD spectra data were achieved via PDA scanning from 190 nm to 500 nm. Betacyanins were monitored only at 535 nm using the above HPLC conditions.

The presence of five BIAs in *C. yanhusuo* was determined by comparing both retention time and spectral data with that of their corresponding authentic standards. The HPLC chromatogram of 5 BIAs was finally monitored at 280 nm. The presence of betalains in *C. yanhusuo* bulbs was judged according to their maximum absorbance at λ 470 nm (betaxanthins) and 535 nm (betacyanins).

## Additional Information

**Accession Code:** The datasets of raw read sequences from each developmental stage and the pooled RNA were deposited in the NCBI Short Read Archive (SRA) database under the study accession SRP026491. The sequences and annotation of our assembled unigenes will be provided if requested.

**How to cite this article**: Liao, D. *et al*. Identification and developmental expression profiling of putative alkaloid biosynthetic genes in *Corydalis yanhusuo* bulbs. *Sci. Rep.* **6**, 19460; doi: 10.1038/srep19460 (2016).

## Supplementary Material

Supplementary Information

Supplementary Dataset 1

Supplementary Dataset 2

Supplementary Dataset 3

Supplementary Dataset 4

Supplementary Dataset 5

Supplementary Dataset 6

Supplementary Dataset 7

Supplementary Dataset 8

Supplementary Dataset 9

## Figures and Tables

**Figure 1:**
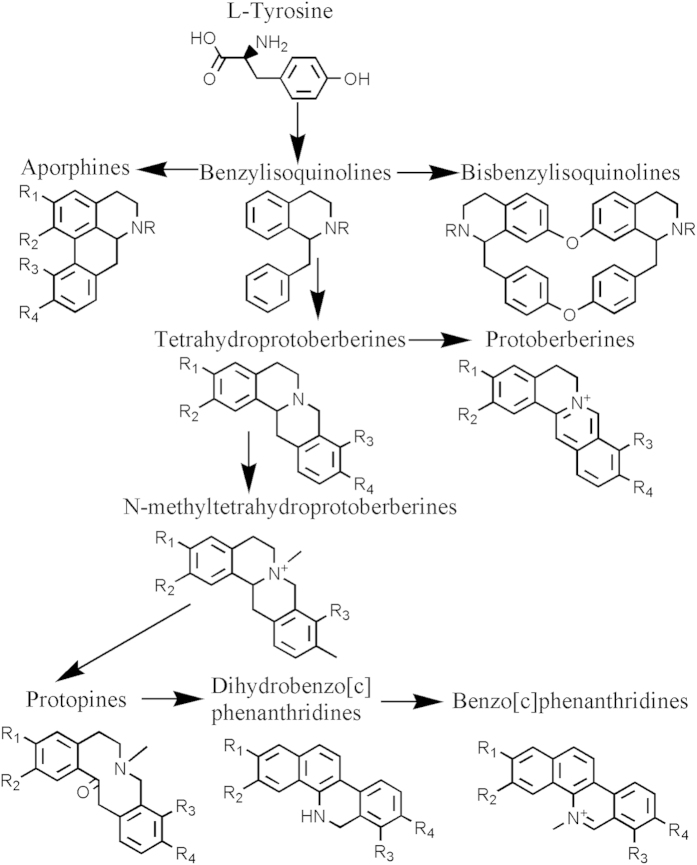


**Table 1:** Summary of *C. yanhusuo* bulb transcriptome data.

Total clean raw reads	25,013,630
Total nucleotides (bp)	2,251,226,700
Q20 percentage	96.22%
GC percentage	44.65%
Average read length (bp)	90
Total contigs	158,101
Length range (bp)	60–5752
Mean contig length (bp)	198
Total scaffolds	65,839
Length range (bp)	100–7977
Mean scaffold length (bp)	340
Total Unigenes	47,081
Total nucleotides (bp)	23,016,560
Length range (bp)	150–7973
Mean unigene length (bp)	489
N50 (bp)	681
Unique mapping reads	9,754,893
No.(%) of annotated unigenes (e < 10^−5^)	30,868 (65.56%)
Nr	30,660 (65.12%)
Swiss-Prot	20,664 (43.89%)
KEGG	13,111 (27.85%)
COG	9,412 (19.99%)
GO	12,455 (26.45%)

**Table 2:** Alignment of Illumina-Solexa 50 bp single end reads onto the *Corydalis* reference transcriptome.

Bulb stage	Day 0	Day 10	Day 30
Total raw reads	7142394	7595670	7043080
Total clean raw reads	7045623 (98.65%)	7504270 (98.80%)	6933847 (98.45%)
Total mapped clean reads	4206094 (59.70%)	4161699 (55.46%)	3112053 (44.88%)
Perfect match	3270224 (46.41%)	3224934 (42.97%)	2383576 (34.38%)
≤2 bp mismatch	935870 (12.28%)	936765 (12.48%)	728477 (10.51%)
Unique match	4199436 (59.60%)	4155601 (55.38%)	3106357 (44.80%)
Multi-position match	6658 (0.09%)	6098 (0.08%)	5696 (0.08%)
Mapped reference genes	45289 (96.19%)	45547 (96.74%)	44831 (95.22%)
Unigenes with RPKM ≥ 2.00	42454(93.74%)	42913(94.22%)	42162(94.05%)
