# Supplementary material for: Identification and developmental expression profiling of putative alkaloid biosynthetic genes in Corydalis yanhusuo bulbs
Source: Sci Rep. 2016 Jan 18;6:19460. doi: 10.1038/srep19460 (PMC4726099; doi:10.1038/srep19460)
Supplement: Supplementary Information [file srep19460-s1.pdf]

Title: Identification and developmental expression profiling of the putative alkaloid biosynthetic genes in *Corydalis yanhusuo* bulbs

Dengqun Liao[1#], Pengfei Wang[1,2#], Chan Jia[1], Peng Sun[1], Jianjun Qi[1], Lili Zhou[1], Xian'en Li[1*]

[1]Institute of Medicinal Plant Development, Chinese Academy of Medical Sciences & Peking Union Medical College, Beijing 100193, PR China

[2] China Pharmaceutical University, Nanjing 210009, China

*Corresponding author:

Xian'en Li

Tel.: +86-10-62810019

Email: xianenli@yeah.net

[#]These authors contributed equally to this work.

Supplementary table and figure legends

Table S1 Known genes involved in plant alkaloid biosynthesis

Table S2 Pathway annotation of *C. yanhusuo* Unigenes

Table S3 The number of differentially expressed Unigenes annotated to KEGG pathways

Table S4 Enriched KEGG pathways for differentially expressed Unigenes in *C. yanhusuo* bulb

Table S5 Enriched biological progresses for differentially expressed Unigenes in *C. yanhusuo* bulb

Table S6 KEGG-mapped alkaloid Unigenes in *C.yanhusuo* bulb transcriptome

Table S7 The list of putative BIAs biosynthetic Unigenes and their expression in *C. yanhusuo* developing bulb

Table S8 The list of putative non-isoquinoline-type alkaloid biosynthetic Unigenes and their expression in *C.yanhusuo* developing bulb

Table S9 BIAs-related OMT homologs in palmatine-producing medicinal plants

Figure S1 Length and gap distribution of Contigs, Scaffolds and Unigenes assembled from Illumina paired-end reads

Figure S2 Length and gap distribution of protein-coding Unigenes predicted by blastx search and ESTScan

Figure S3 COG classification of *C. yanhusuo* Unigenes

Figure S4 GO classification of *C. yanhusuo* Unigenes

Figure S5 Distribution of the coverage of the Unigenes in libraries Day0, Day10 and Day30

Figure S6 COG classification of *C. yanhusuo* Unigenes differentially expressed during the bulb development

Figure S7 The UV-VIS spectra of *C.yanhusuo* peaks 1-7 detected at 470nm

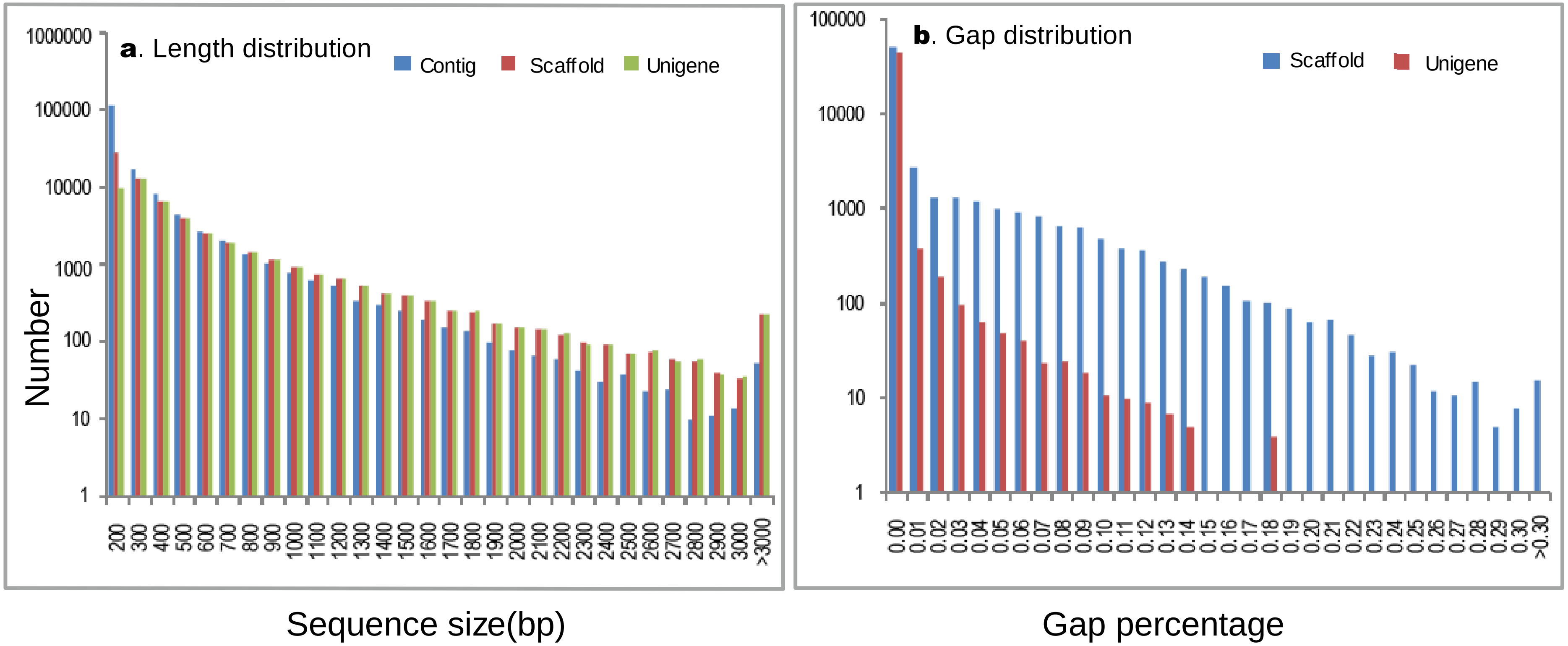

Figure S1 Length and gap distribution of Contigs, Scaffolds and Unigenes assembled from Illumina paired-end reads

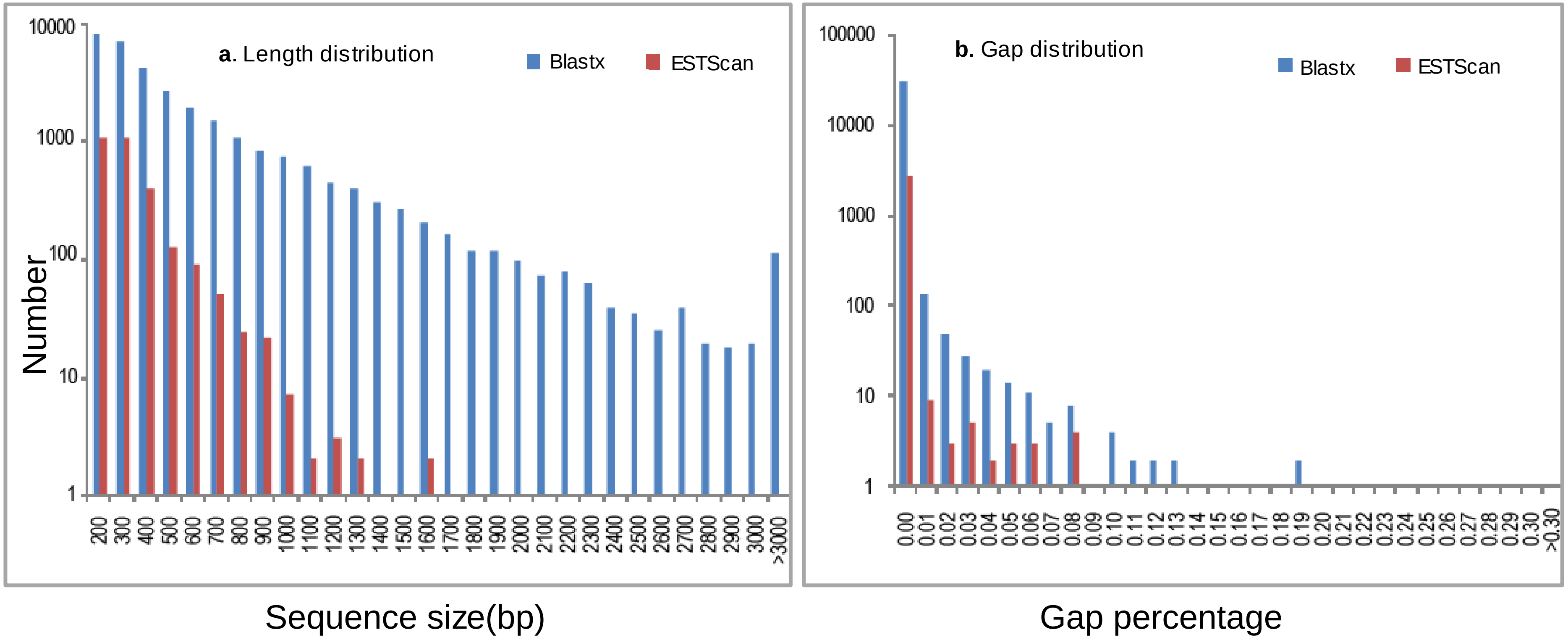

Figure S2 Length and gap distribution of protein-coding Unigenes predicted by Blastx search and ESTScan

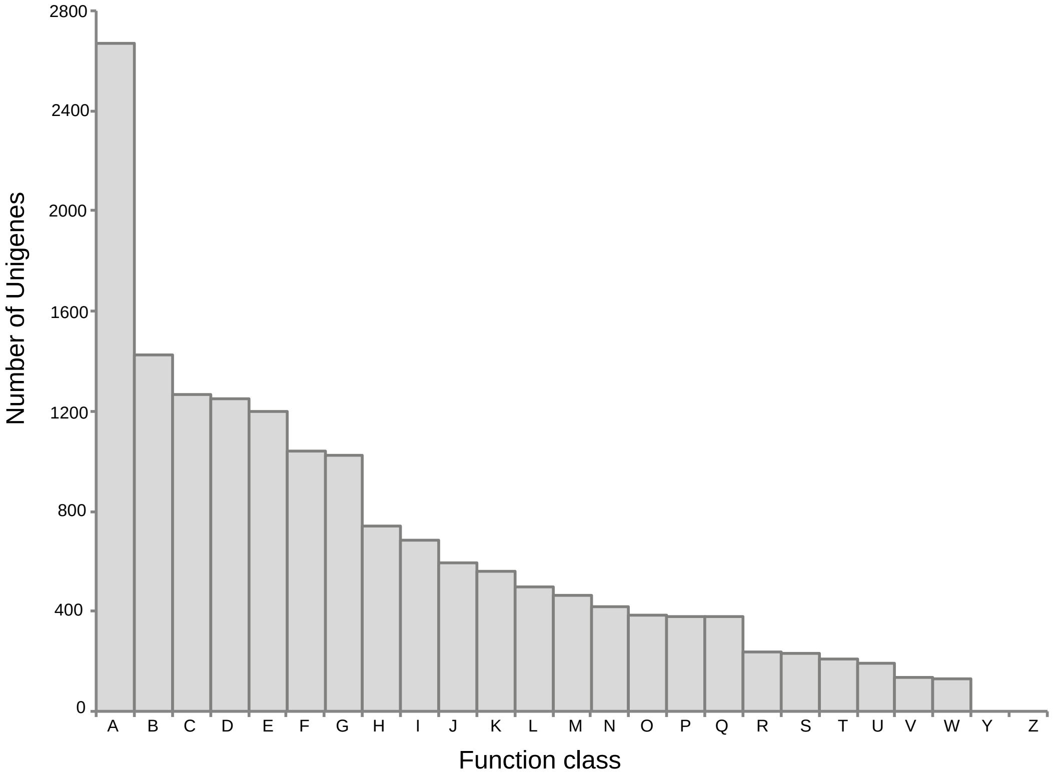

Figure S3 COG classification of *C. yanhusuo* Unigenes

A. General function prediction only; B. Transcription; C. Posttranslational modification, protein turnover, chaperones; D. Replication, recombination and repair ; E. Translation, ribosomal structure and biogenesis; F. Carbohydrate transport and metabolism; G.Signal transduction mechanisms; H. Amino acid transport and metabolism; I. Function unknown; J. Cell wall/membrane/envelope biogenesis; K . Energy production and conversion; L. Cell cycle control, cell division, chromosome partitioning; M. Inorganic ion transport and metabolism; N. Secondary metabolites biosynthesis, transport and catabolism; O. Intracellular trafficking, secretion, and vesicular transport; P. Coenzyme transport and metabolism; Q. Lipid transport and metabolism; R. Defense mechanisms; S. Cytoskeleton; T. Chromatin structure and dynamics; U. Nucleotide transport and metabolism; V. Cell motility; W. RNA processing and modification; Y. Extracellular structures; Z. Nuclear structure

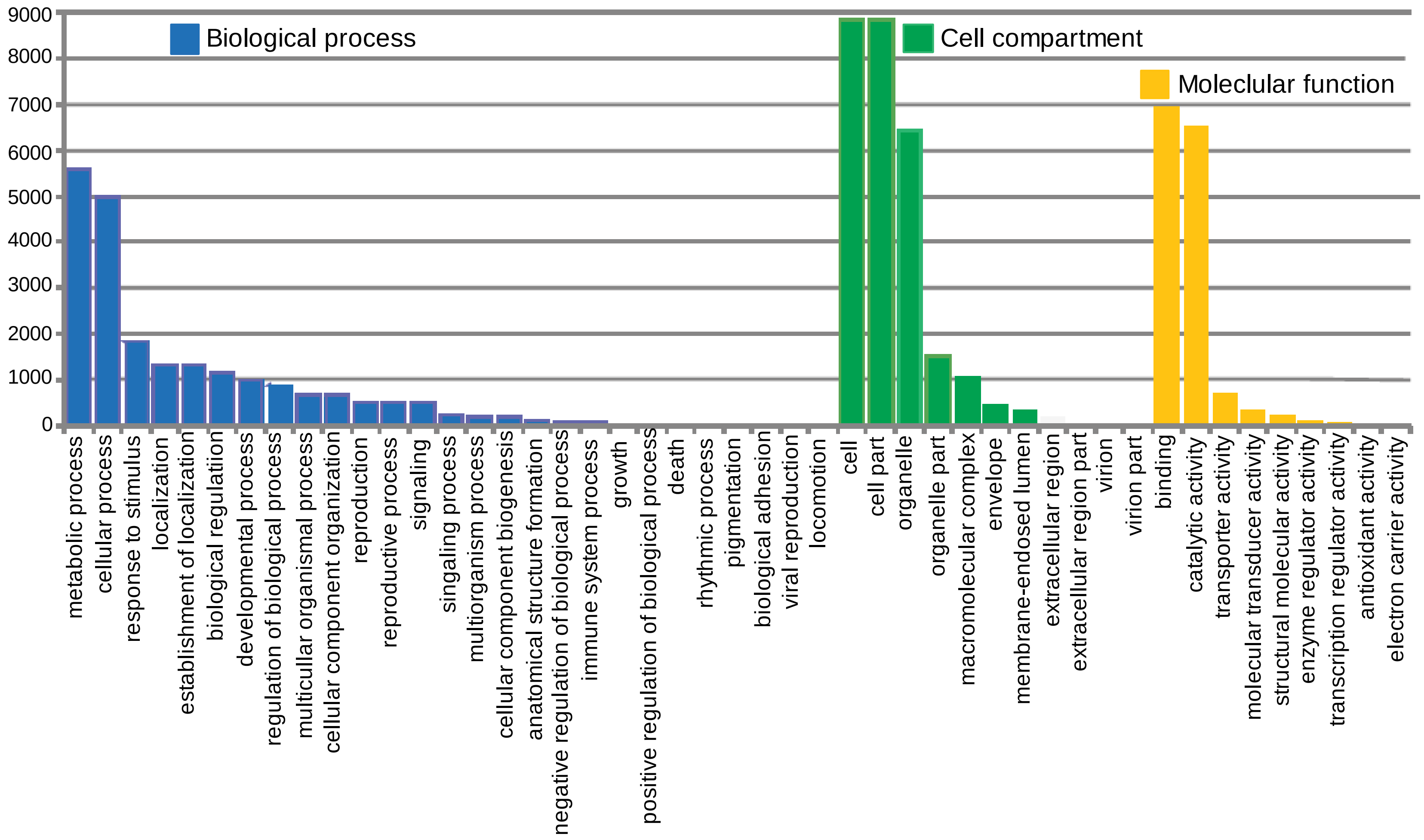

Figure S4 GO classification of *C. yanhusuo* Unigenes

X-axis shows Go category at level 2, Y-axis shows Number of Unigenes assigned to each category.

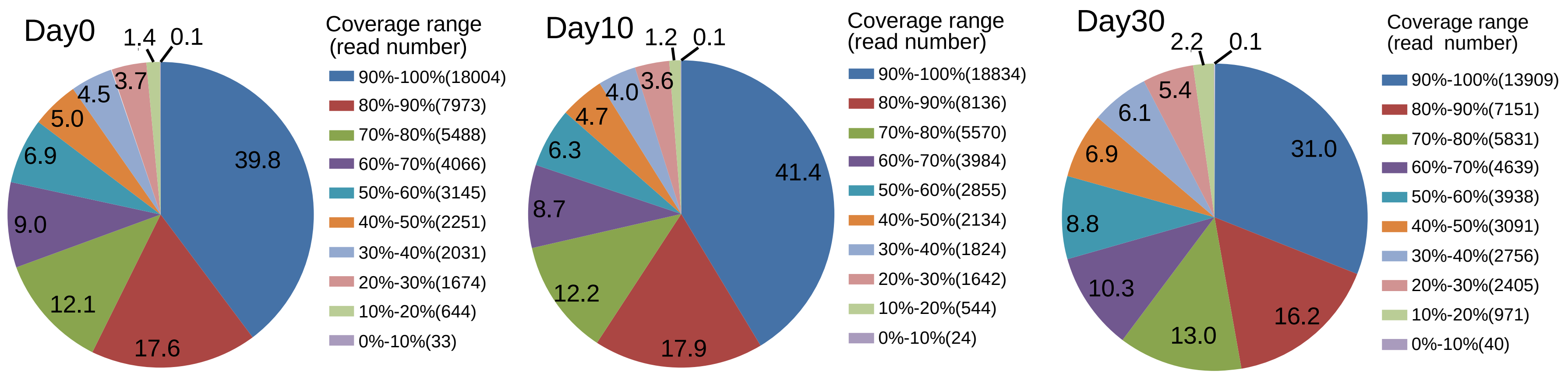

Figure S5 Distribution of the coverage of the Unigenes in libraries Day0, Day10 and Day30

The coverage of a Unigene meant the percentage of a Unigene covered by reads in each library and was calculated by the ratio of the base number in a Unigene covered by unique mapping reads to the total bases number of that Unigene.

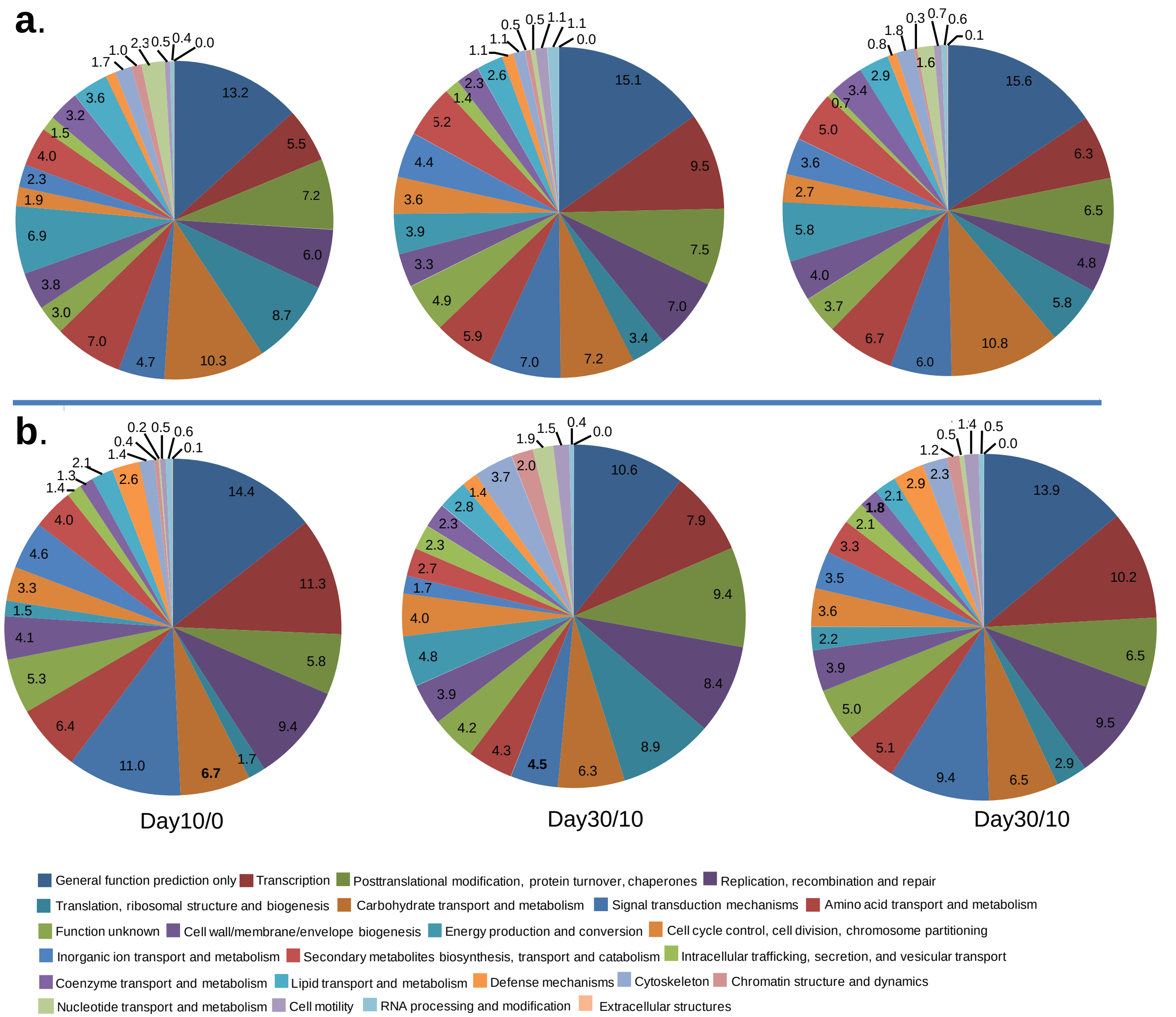

FigureS6 COG classification of *C. yanhusuo* Unigenes differentially expressed during the bulb development presented is percentage of up- (**a.**) and down- (**b.**) regulated Unigenes in each comparison to individual COG category.

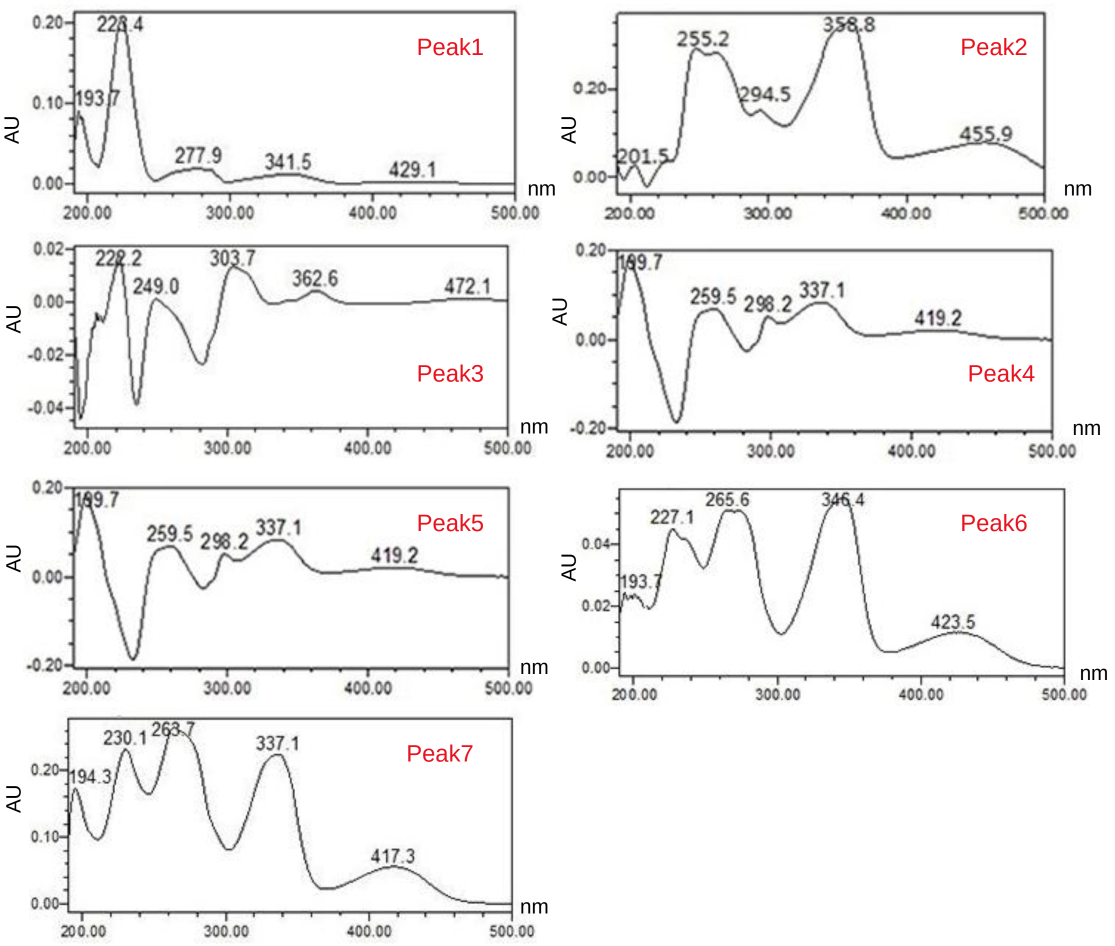

Figure S7 The UV-VIS spectra of *C.yanhusuo* peaks 1-7 detected at 470nm
